# Growth Outcomes and Dietary Strategies in Non-IgE-Mediated Food Allergies

**DOI:** 10.3390/nu18142387

**Published:** 2026-07-22

**Authors:** Gianluca Di Cesare, Chiara Monachesi, Annalisa Carciofi, Francesca Borgiani, Anna Rita Incampo, Simona Gatti, Elena Lionetti

**Affiliations:** 1 Department of Maternal and Child Health, Azienda Ospedaliero-Universitaria delle Marche, 60123 Ancona, Italy; gianluca.dicesare@ospedaliriuniti.marche.it; 2 Department of Pediatrics, Polytechnic University of Marche, 60123 Ancona, Italy; c.monachesi@pm.univpm.it (C.M.); annalisa.carciofi@ospedaliriuniti.marche.it (A.C.); francesca.borgiani@ospedaliriuniti.marche.it (F.B.); a.r.incampo@pm.univpm.it (A.R.I.); m.e.lionetti@staff.univpm.it (E.L.)

**Keywords:** Non–IgE-mediated food allergies, dietary management, CMA, growth, BMI, FPE, FPIAP

## Abstract

Background/Objective: Non-IgE-mediated food allergies (non-IgE-FAs) are increasingly recognized in infancy as causes of delayed gastrointestinal symptoms, nutritional compromise, and impaired growth. This retrospective study aimed to evaluate longitudinal growth outcomes in children with non-IgE-FAs managed with different dietary interventions and to explore phenotype-specific differences and clinical correlations. Methods: Children diagnosed with non-IgE-FAs at the Pediatric Clinic Unit of Salesi Hospital (Ancona, Italy) between July 2022 and June 2025 were included. Anthropometric, clinical, and laboratory data were collected at baseline (T0) and follow-up visits at 6, 12, and 18 months. Weight-, length-, and BMI-for-age z-scores were calculated using Growth Calculator 4.0. Longitudinal growth trends were assessed through individual linear regression slopes, and dietary intervention effects were evaluated with ANOVA models. Results: Thirty-seven children were included (57% male), 95% of whom were diagnosed with CMA. Slope analyses demonstrated significant positive trends for BMI z-scores (median 0.340, IQR −0.171 to 1.130; *p* = 0.013). BMI slope differed according to phenotype when comparing children with FPE and those with FPIAP (mean slope = 0.068 ± 0.795 vs. 0.782 ± 0.873, respectively; *p* = 0.048). No significant differences in BMI improvement were observed between children on an elimination diet, breastfed children whose mothers followed an elimination diet, and those on a free diet. Conclusion: Dietary management in children with non-IgE-FAs was associated with improved growth trajectories, particularly in BMI-for-age z-scores. Children with FPE showed a less favorable BMI trajectory than those with FPIAP. Although the choice of dietary strategy did not significantly affect growth outcomes in this cohort, individualized nutritional assessment and judicious use of elimination diets remain essential. Larger prospective studies are needed to refine phenotype-specific nutritional strategies and optimize long-term outcomes.

## 1. Introduction

Non-IgE-mediated food allergies represent a substantial proportion of pediatric food allergies, which are characterized by delayed gastrointestinal symptoms and frequently overlap with other pediatric conditions, often leading to diagnostic delay. 

The spectrum of non-IgE-mediated food allergy encompasses well-defined clinical entities, including Food Protein-Induced Enterocolitis Syndrome (FPIES), Eosinophilic Esophagitis (EoE), Food Protein-Induced Enteropathy (FPE), and Food Protein-Induced Allergic Proctocolitis (FPIAP), as well as more controversial conditions grouped under food protein-induced dysmotility disorders (FPIDD), such as food protein-induced gastroesophageal reflux disease (FPGORD) and food protein-induced constipation (FPC) [1,2].

Persistent exposure to the triggering allergen may result in chronic inflammation, malabsorption, and feeding difficulties, which together can negatively influence growth and nutritional status. The risk of growth and nutritional impairment is particularly evident at the time of diagnosis. The severity of growth alterations differs across phenotypes, with FPIES showing the greatest burden, mainly in severe or multi-trigger cases. With regard to FPE, patients have been reported to exhibit the highest prevalence of wasting and underweight at the time of diagnosis. This may be explained by inflammation and mucosal injury triggered by allergic reactions to food antigens, which can impair nutrient absorption in the gastrointestinal tract, thereby contributing to nutritional deficiencies and, ultimately, growth impairment. By contrast, FPIAP is often considered a milder disorder with preserved growth in most infants [3].

Importantly, prospective evidence demonstrates that early, structured dietary counseling delivered by specialized pediatric dietitians can significantly improve anthropometric outcomes and reduce malnutrition rates. These findings highlight nutritional management as a cornerstone of care, underscoring the need for individualized, age-specific, and allergen-specific nutritional monitoring. Moreover, they emphasize the necessity for further longitudinal and controlled studies to better define optimal management strategies and improve growth outcomes in this population [3,4].

In this study, we assessed growth outcomes among children with non-IgE-FAs managed with different dietary interventions. More specifically, the primary outcome was to evaluate growth in children undergoing an elimination diet and/or exclusively breastfed by mothers adhering to an elimination diet. The secondary outcomes were:

- To determine whether different dietetic strategies are associated with distinct clinical outcomes;
- To explore potential correlations between specific non-IgE-mediated food allergy phenotypes and clinical outcomes.

## 2. Materials and Methods

This retrospective single-center observational study included all patients evaluated in the outpatient setting at the Pediatric Clinic Unit (UOC Clinica Pediatrica) of Salesi Hospital, Ancona, between 1 July 2022, and 30 June 2025, who were subsequently diagnosed with non-IgE-FAs [5]. 

Patients with fewer than two outpatient visits were excluded, as at least two assessments were required to evaluate changes in growth over time. Additional exclusion criteria were considered a diagnosis of eosinophilic esophagitis, which was excluded as a distinct clinicopathological entity characterized by mixed immunological mechanisms, and FPIDD, which were excluded because of diagnostic uncertainty and their frequent overlap with other gastrointestinal conditions.

Data were collected according to an internal standardized protocol routinely used for the clinical assessment and follow-up of outpatients at the Pediatric Clinic Unit. Although follow-up schedules reflected routine clinical practice and were therefore not identical for all patients, the majority had complete data at baseline and at the first follow-up visit (approximately 6 months). Consequently, analyses comparing predefined time points were primarily based on these assessments. For the evaluation of longitudinal growth trajectories, individual slope analyses were performed using all available anthropometric measurements for each patient, allowing the inclusion of participants with different numbers and timing of follow-up visits. All patients received standardized nutritional counseling from a specialized pediatric nutritionist at each follow-up visit. Information was extracted from the electronic medical record system (SICO). Eligible patients evaluated in the previously mentioned period were identified using the following keywords: “CMA,” “procto,” “protein,” and “FPIES.”

In addition to baseline demographic characteristics including gender and age, anthropometric parameters, biochemical measures, clinical signs and symptoms, diagnoses, and dietary interventions were recorded. Assessments were performed at four predefined time points: baseline (T0) and follow-up visits at 6 (T6), 12 (T12), and 18 months (T18). T0 corresponded to the timing of diagnostic confirmation, while T6, T12, and T18 represented follow-up evaluations.

Z-scores for weight-for-age, length-for-age, and BMI-for-age were calculated using the Growth Calculator 4.0 software [3].

Descriptive statistics were used to summarize the main characteristics of the study population. Continuous variables were reported as mean ± standard deviation (SD) for normally distributed data or median and interquartile range (IQR) for non-normally distributed data, as appropriate based on data distribution. The normality of continuous variables was assessed using the Shapiro–Wilk test. Categorical variables were expressed as frequencies and percentages. For comparisons between independent groups, continuous variables were analyzed using the independent samples *t*-test for normally distributed data and the Mann–Whitney U test for non-normally distributed data. For within-subject comparisons, the paired samples *t*-test was used for normally distributed variables, while the Wilcoxon signed-rank test was applied for non-normally distributed data. Associations between categorical variables were evaluated using the chi-square test. For paired categorical data, the McNemar test was used to assess changes over time or differences between matched observations. Changes in growth parameters were evaluated both as the absolute difference between baseline and follow-up z-scores and as the individual slope of change over time, calculated using linear regression across time points. The mean slope was then compared against zero using a one-sample Wilcoxon signed-rank to determine whether a significant overall growth trend was present during the study period. Differences in continuous outcome changes between independent groups were assessed using one-way ANOVA when more than two groups were compared. When appropriate, post hoc pairwise comparisons were performed using the Games-Howell or Tukey test. The effects of two categorical factors and their interaction on continuous outcome changes were assessed using a two-way ANOVA. Because most allergen reintroductions occurred after the 6-month follow-up visit, the analysis was restricted to the first 6 months of follow-up to minimize the potential confounding effect of dietary reintroduction on growth outcomes. A *p*-value < 0.05 was considered statistically significant. Statistical analyses were performed using R software version 4.3.1.

## 3. Results

A total of 37 patients were included in the final analysis, of whom 57% were male. Baseline characteristics, including clinical signs and symptoms, laboratory findings, non-IgE-mediated food allergy phenotypes, and corresponding dietary interventions, are summarized in Table 1.

Cow’s milk allergy (CMA) was diagnosed in 95% of patients (35/37). Among these, one patient also had a concomitant soy allergy, and another had wheat allergy. Of the remaining patients (5%, 2/37), one had wheat allergy and the other fish allergy.

**Figure 1:**
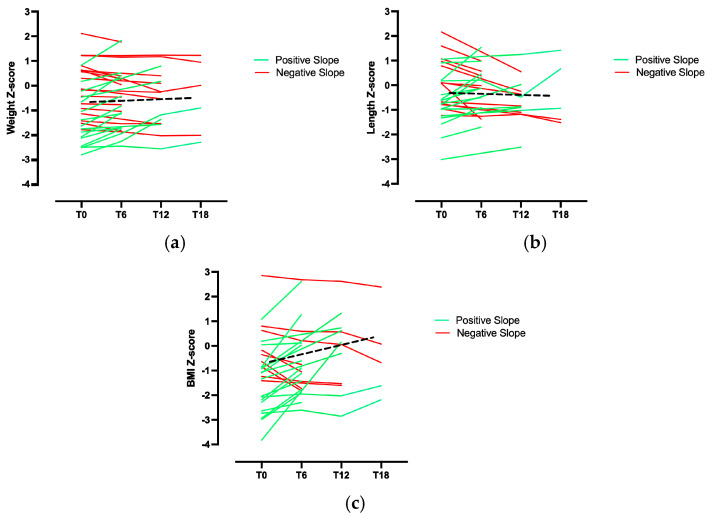
Growth Trajectories: (**a**) individual slopes for weight-for-age z-score; (**b**) individual slopes for length-for-age z-score; (**c**) individual slopes for BMI-for-age z-score. A simple linear regression analysis was performed to assess the linear trend (dashed line).

Paired comparisons between baseline and follow-up z-scores did not show statistically significant differences. However, when longitudinal trends were evaluated using individual slopes derived from linear regression across available multiple time points (T0, T6, T12, T18), significant positive trends were observed when the mean slopes were tested against zero using a one-sample *t*-test (Figure 1). 

The analysis of individual slopes demonstrated a significant positive trend over time for BMI-for-age z-scores (median 0.340, IQR −0.171 to 1.130; *p* = 0.013), indicating an overall improvement in this growth parameter during the study period. No significant overall trend was observed for the other growth indicators.

**Figure 2:**
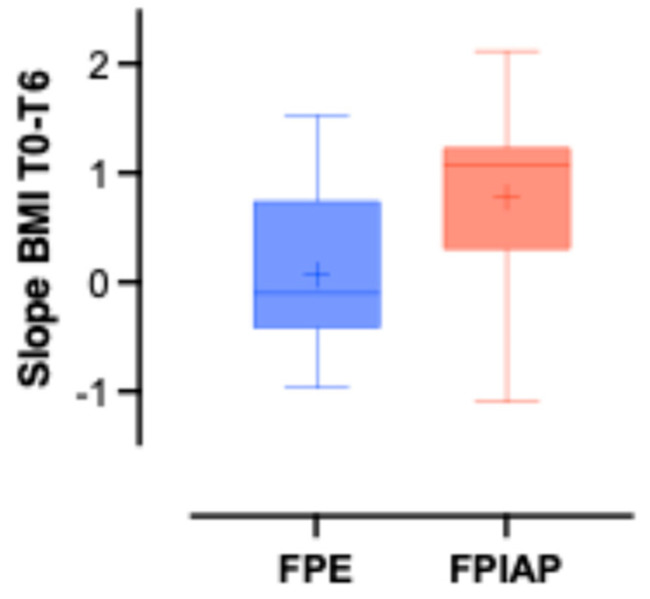
Comparison of the mean slope values between categories.

Baseline growth parameters were compared among allergy phenotypes using one-way ANOVA. No significant differences were observed among phenotypes in weight-for-age, length-for-age, and BMI-for-age z-scores at baseline. In contrast, BMI slope differed according to phenotype when comparing children with FPE and those with FPIAP (mean slope = 0.068 ± 0.795 vs. 0.782 ± 0.873, respectively; *p* = 0.048). This difference was associated with a large standardized effect size (Cohen’s d = 0.851) (Figure 2). Due to the small sample size of the FPIES subgroup, this group was excluded from post hoc pairwise comparisons. 

**Figure 3:**
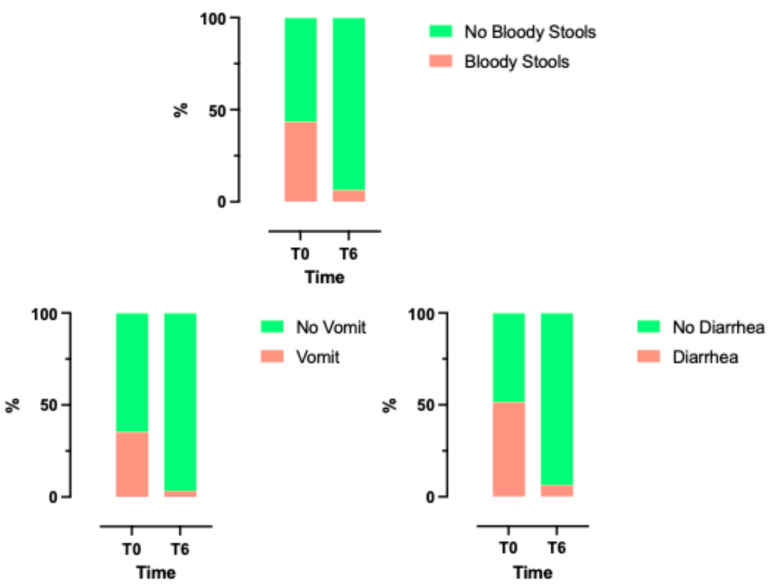
Changes in the prevalence of bloody stools, vomiting, and diarrhea from baseline (T0) to 6-month follow-up (T6).

At baseline, 35% of children presented with vomiting, 51% with diarrhea, and 43% with bloody stools. The prevalence of these symptoms decreased during follow-up, reaching 3%, 6%, and 6%, respectively, at T6. Constipation and atopic dermatitis showed less consistent patterns, decreasing from 11% at baseline to 3% at follow-up and from 19% to 6%, respectively. When comparing symptom prevalence between baseline (T0) and 6-month follow-up (T6), a significant reduction was observed for vomiting (*p* < 0.001), diarrhea (*p* < 0.001) and bloody stools (*p* = 0.004) (Figure 3). Symptom resolution was observed in the majority of cases for vomiting (12/13; 92%), diarrhea (16/17; 94%) and bloody stools (11/12; 92%). No statistically significant changes were found for constipation and atopic dermatitis.

A two-way ANOVA was conducted to evaluate the effects of maternal elimination diet, child elimination diet, and their interaction on growth parameters over a 6-month follow-up period, but no significant effects were observed for weight-for-age, length-for-age, or BMI-for-age z-score changes (all *p* > 0.05). However, the small and markedly unbalanced factorial cell sizes limited the precision and statistical power of these exploratory analyses. No additional follow-up period was evaluated, as the eliminated allergen had already been reintroduced in the majority of patients at 18 months (72%). 

## 4. Discussion

This retrospective study examined the clinical and growth outcomes of children with non-IgE-FAs, with particular attention to longitudinal trajectories across phenotypes and the effects of different dietary interventions. CMA was diagnosed in nearly all participants, confirming cow’s milk protein as the predominant trigger of non-IgE-FAs in infancy [6].

The primary aim of this study was to assess growth trajectories throughout the follow-up period. Our findings indicate that improvements in BMI-for-age z-scores may serve as an early and sensitive marker of clinical response to dietary management. In contrast, meaningful changes in weight-for-age and length-for-age z-scores may require longer observation, as catch-up growth typically occurs more gradually. These results highlight the value of extended growth monitoring in children with non-IgE-FAs. However, given the retrospective observational design of the study, these findings should be interpreted with caution. Although the observed improvement was temporally associated with dietary management, information on potentially relevant nutritional factors, including total caloric intake and macronutrient distribution, was not systematically available, precluding adjustment for these variables. In contrast, changes in weight and linear growth may become apparent only over a longer follow-up period, as catch-up in weight and length typically requires more time to become evident. These observations underscore the importance of extended monitoring when evaluating growth trajectories in children with non-IgE-mediated food allergies.

The study also explored growth differences among non-IgE-mediated food allergy phenotypes. Although the exclusion of the FPIES subgroup from comparative analyses represents a limitation of the current cohort, it also reflects the relative rarity of this phenotype. 

The observed difference in BMI-for-age z-score trajectories between children with FPIAP and those with FPE is clinically noteworthy. Children with FPIAP exhibited a greater improvement in BMI-for-age z-score over time than those with FPE, with a large standardized effect size. Although this finding should be interpreted cautiously given the limited sample size, it is consistent with the distinct pathophysiological features of FPE, which is characterized by chronic intestinal inflammation, mucosal injury, and malabsorption. These mechanisms may result in greater nutritional compromise and a slower recovery of growth compared with more localized conditions such as FPIAP [4]. The observed growth deficit in children with FPE provides further insight into phenotype-specific growth patterns and suggests that this phenotype may be associated with a greater nutritional burden than FPIAP. Interestingly, our results differ slightly from those previously reported by Coppola et al., who identified baseline differences in length-for-age z-scores between the two phenotypic groups [4].

With regard to the clinical manifestations of non-IgE-mediated food allergies, gastrointestinal symptoms tend to resolve in the majority of cases within approximately six months. While this may reflect an optimal response to dietary management, it may also indicate the spontaneous improvement of benign conditions such as FPIAP. Indeed, recent studies have suggested that a “watch-and-wait” approach may be reasonable before initiating an elimination diet in selected cases [7]. However, the lack of consistent randomized controlled trials limits the strength of evidence supporting this strategy. In our center, this approach is rarely applied, as only 5% of patients did not undergo either a direct elimination diet or a maternal elimination diet during breastfeeding.

An additional objective of this study was to explore the effects of maternal elimination diets in breastfed infants and direct elimination diets in children on growth outcomes. In this cohort, no statistically detectable differences in growth trajectories were observed between elimination-diet strategies; however, the study was not powered to exclude clinically meaningful differences. Therefore, individualized nutritional assessment, the careful indication of elimination diets and the support from a pediatric nutrition specialist remain highly relevant [8].

The present analysis was conducted on a relatively small sample, particularly within phenotype subgroups, partly reflecting the inherent diagnostic challenges of non-IgE-mediated food allergies. Consequently, the study should be regarded as exploratory, and the limited sample size may have reduced the statistical power to detect differences across phenotypes and dietary strategies, thereby limiting the generalizability of the findings. In addition, the absence of a healthy control group or a comparison cohort of children with IgE-mediated food allergies limits the ability to distinguish the effects of dietary management from those of other aspects of routine clinical care. Furthermore, the single-center retrospective design may have introduced selection bias and confounding by indication, as children with more severe disease may have been preferentially managed with more restrictive dietary strategies, including extensively hydrolyzed or amino acid-based formulas and multiple-food elimination diets. Multiple statistical comparisons were performed without adjustment for multiplicity. Therefore, subgroup analyses should be regarded as exploratory and hypothesis-generating, and require confirmation in larger prospective multicenter studies. Despite these limitations, the longitudinal design provides valuable insight into growth trajectories over time, particularly in BMI-for-age z-scores, and highlights potential phenotype-specific differences in growth outcomes. Future prospective multicenter studies with larger cohorts and extended follow-up are warranted to validate these findings. The application of linear mixed-effects models would provide a more robust framework for analyzing longitudinal growth trajectories by accommodating repeated measurements, missing data, and time-varying covariates, such as age and symptom severity. Such studies may also help identify determinants of growth recovery and further refine dietary management strategies in children with non-IgE-mediated food allergies.

## 5. Conclusions

Non-IgE-mediated food allergies are increasingly recognized as important causes of gastrointestinal symptoms and growth impairment in infancy, with cow’s milk protein representing the most common trigger. In this retrospective observational study, children managed with dietary interventions showed an overall improvement in growth trajectories, particularly in BMI, which emerged as an early and sensitive marker of clinical response. Growth differences among phenotypes were also observed, with children affected by FPE exhibiting greater impairment in growth, likely reflecting the increased nutritional burden associated with chronic intestinal inflammation and malabsorption. The choice of dietary strategy does not appear to significantly affect growth outcomes; nevertheless, the importance of individualized nutritional strategies and the careful indication of elimination diets remain highly relevant [9]. Although limited by the small sample size and diagnostic heterogeneity, these findings underscore the potential importance of timely recognition, structured dietary counseling, and long-term growth monitoring. Further prospective, larger-scale studies are needed to better define phenotype-specific outcomes and optimize nutritional management in this population.

## Figures and Tables

**Table 1:** Baseline characteristics of the study population.

Parameter (N = 37 *)	Value
Males (%)	57
Age (T0) (months)	19.14 ± 26.02
Weight (T0) (kg)	8.82 ± 4.03
Weight (T0) (SDS)	−0.72 ± 1.29
Length (T0) (cm)	75.71 ± 14.48
Length (T0) (SDS)	−0.27 ± 1.21
BMI (T0) (kg/m^2^)	14.84 ± 2.19
BMI (T0) (SDS)	−0.77 ± 1.45
Hb (T0)	11.14 ± 1.37
Hb normal (T0) (%)	63
Ferritin (T0)	30.90 ± 33.09
Ferritin normal (T0) (%)	50
Eosinophils (T0)	706 ± 293.56
Eosinophils normal (T0) (%)	40
Atopic dermatitis (T0) (%)	19
Vomiting (T0) (%)	35
Diarrhea (T0) (%)	51
Constipation (T0) (%)	11
Bloody stools (T0) (%)	43
FPIAP (%)	43
FPIES (%)	16
FPE (%)	41
EHF (T0) (%)	24
AAF (T0) (%)	0
HRF (T0) (%)	11
Elimination diet—Mother (T0) (%)	11
Elimination diet—Child (T0) (%)	54
Elimination diet—Mother + Child (T0) (%)	30
No Diet (T0) (%)	5

* Four patients had four follow-up visits; six patients had three follow-up visits; the remaining patients had two follow-up visits.

## Data Availability

The data presented in this study are available on request from the corresponding author due to privacy restrictions.

## Abbreviations

The following abbreviations are used in this manuscript:

FA	food allergy
CMA	cow’s milk allergy
FPE	food protein-induced enteropathy
FPIAP	food protein-induced allergic proctocolitis
FPIES	Food Protein-Induced Enterocolitis Syndrome
EoE	eosinophilic oesophagitis
FPIDD	food protein-induced dysmotility disorders
FPGORD	food protein-induced gastroesophageal reflux disease
FPC	food protein-induced constipation
SD	standard deviation
IQR	interquartile range
BMI	body mass index
